# Perceived relative harm of electronic cigarettes over time and impact on subsequent use. A survey with 1-year and 2-year follow-ups

**DOI:** 10.1016/j.drugalcdep.2015.10.014

**Published:** 2015-12-01

**Authors:** Leonie S. Brose, Jamie Brown, Sara C. Hitchman, Ann McNeill

**Affiliations:** a Department of Addictions, Institute of Psychiatry, Psychology and Neuroscience, King's College London, 4 Windsor Walk, London SE5 8BB, UK; b UK Centre for Tobacco and Alcohol Studies (UKCTAS), UK; c Department of Clinical, Educational and Health Psychology, University College London, 1–19 Torrington Place, London WC1E 7HB, UK

**Keywords:** Nicotine, Tobacco, Electronic cigarettes, Harm, Behavior

## Abstract

**Introduction:**

Media presentations of e-cigarettes may affect perception of the devices which may influence use.

**Objectives:**

To assess in a cohort of past-year smokers (1) if perceived harm of e-cigarettes relative to cigarettes changed over time, (2) predictors of perceived relative harm, (3) if perceived relative harm predicted subsequent e-cigarette use among never-users.

**Methods:**

Longitudinal web-based survey of a general population sample of British smokers and ex-smokers, waves in 2012 (*n* = 4553), 2013 and 2014 (44%, 31% response rate, respectively). Changes over time were assessed using Friedman and McNemar tests, *n* = 1204. Perceived relative harm at wave 3 was regressed onto perceived relative harm at waves 1 and 2, while adjusting for socio-demographics and change in smoking and e-cigarette status, *n* = 1204. Wave 2 e-cigarette use among 1588 wave 1 never-users was regressed onto wave 1 socio-demographics, smoking status and perceived relative harm.

**Results:**

Perceived relative harm changed (*χ*^2^ = 20.67, *p* < 0.001); the proportion perceiving e-cigarettes to be less harmful than cigarettes decreased from 2013 to 2014 (*χ*^2^ = 16.55, *p* < 0.001). Previous perception of e-cigarettes as less harmful, having tried e-cigarettes and having stopped smoking between waves predicted perceiving e-cigarettes as less harmful than cigarettes. Perceiving e-cigarettes to be less harmful than cigarettes predicted subsequent use, adjusting for other characteristics (OR = 1.39; 95% CI: 1.08–1.80, *p* = 0.011).

**Conclusion:**

Among a cohort of smokers and ex-smokers, accurately perceiving e-cigarettes as less harmful than smoking predicted subsequent e-cigarette use in never-users; this perception declined over time. Clear information on the relative harm of cigarettes and e-cigarettes is needed.

## Highlights

• A cohort of smokers and ex-smokers was followed over a period of two years.
• Perceived harm of electronic cigarettes relative to cigarettes increased over time.
• Smoking cessation ande-cigarette use predicted subsequent perceived relative harm.
• Perceived relative harm predicted subsequent use of e-cigarettes in non-users.

## 1 Introduction

Combustible tobacco cigarettes (referred to as cigarettes in the remainder of this article) kill between half and two thirds of continuing smokers (Banks et al., 2015, Doll et al., 2004). It is primarily the nicotine in cigarettes that produces the addiction to tobacco but most of the health harms of smoking are related to other components of cigarette smoke (Benowitz, 2009). Electronic cigarettes (e-cigarettes) deliver nicotine without burning tobacco. While the long-term health effects of e-cigarettes are as yet unknown and may differ across brands, types and usage (Britton and Bogdanovica, 2014, Cheng, 2014, Goniewicz et al., 2014, Kosmider et al., 2014), a group of experts with expertise in nicotine and tobacco research from different disciplines estimated that e-cigarettes are likely to be at least 95% less harmful than cigarettes (Nutt et al., 2014). Whilst the exact figure is still to be determined, most experts agree that continued smoking of cigarettes will be more harmful to an individual's health than using e-cigarettes (Farsalinos and Polosa, 2014, Grana et al., 2014). The potential harms and benefits and appropriate regulation of e-cigarettes are being publically discussed extensively among experts (Letter from 56 Specialists, 2014, Letter from 129 Signatories, 2014, McNeill et al., 2014). These discussions are not limited to the scientific community; many media reports cover e-cigarettes, and although no reliable data are available, reports may often focus on rising prevalence of use, explosions or poisoning linked to them (for example Meikle, 2014, BBC, 2014, BBC, 2015). Media reports, increased use (McMillen et al., 2014, Richardson et al., 2014a) and advertising (Bauld et al., 2014, McCarthy, 2014, Richardson et al., 2014a) may affect perceptions of the relative harm of e-cigarettes, particularly in the absence of equally intense discussion of the enormous health harms of cigarettes.

Perceived harm or perceived risk influence behaviour and in the field of smoking, associations between harm perception and use have for example been reported for nicotine replacement therapy and smokeless tobacco (O’Connor et al., 2007, Shiffman et al., 2008). However, this association has not consistently been found; one study of adult smokers in England reported no association between perceived harm of long-term nicotine replacement therapy and reported use (Black et al., 2012).

Cross-sectional studies have found associations between lower perceived harm of e-cigarettes and e-cigarette use (Adkison et al., 2013, Amrock et al., 2015, Pokhrel et al., 2015, Richardson et al., 2014b). However, no studies have documented whether perceptions of e-cigarette harm prospectively predict e-cigarette use. Because previous use may affect harm perception, it is important to use longitudinal data to assess whether harm perceptions influence use among those who have never previously used e-cigarettes. Additionally, because of the public debate and insofar as perceptions are associated with use, it is important to track perceptions of e-cigarettes over time and to assess socio-demographic and smoking predictors of those perceptions.

This study had three specific aims. First, to assess whether the perceived harm of e-cigarettes relative to cigarettes changed over a two-year period (2012 to 2014) in a cohort of smokers and ex-smokers. Second, to assess predictors of perception of e-cigarettes as less harmful than cigarettes; and third, to assess whether perceived relative harm in 2012 predicted subsequent e-cigarette use in 2013 among respondents who had never previously used an e-cigarette while adjusting for demographics and smoking status.

## 2 Methods

### 2.1 Design and sample

We used data from a longitudinal web-based survey of a national general population sample of smokers and ex-smokers (past year at baseline) in Great Britain. Members of an online panel managed by Ipsos MORI were invited to participate in a survey about smoking. Those who accepted (*n* = 23,785) were screened and past-year smokers (*n* = 6165) were eligible for the survey. Quotas were imposed to ensure broad representativeness by sex, age, and region. Wave 1 (November/December 2012) was completed by 5000 respondents, of whom 4553 were aware of e-cigarettes. Of those aware of e-cigarettes at wave 1, *n* = 2011 respondents (44.2%) completed wave 2 in December 2013 and *n* = 1407 (30.9%) wave 3 in December 2014. Wave 1 sample characteristics were broadly similar to those of representative samples from a household survey (Brown et al., 2014, Fidler et al., 2011). Wave 1 characteristics including perceived relative harm have been described previously (Brown et al., 2014) and data from waves 1 and 2 have been used to assess associations of e-cigarette use with changes in smoking behaviour (Brose et al., 2015, Hitchman et al., 2015). Overall, 1217 respondents were aware of e-cigarettes throughout and rated their perceived relative harm at all three waves.

To address aims 1 and 2, thirteen respondents who were unsure at any wave about their smoking status or whether they had tried e-cigarettes were excluded, leaving 1204 respondents for analysis. To address aim 3, 416 wave 1 users and seven who were unsure about their smoking or whether they had tried e-cigarettes were excluded, leaving data from 1588 respondents who were not using e-cigarettes at wave 1 and were followed up at wave 2. Secondary analysis included 364 respondents who had never used e-cigarettes at wave 2 and were followed up at wave 3.

### 2.2 Measures

Demographics included age (continuous, for main analyses grouped as 18 to 24; 25 to 39; 40 to 54; 55 and over), gender (male; female), education (collapsed into: no higher education; some higher education; don’t know/prefer not to say) and annual household income (collapsed into: ≤£30,000; >£30,000; don’t know/prefer not to say).

At each wave, participants were asked if they had ever tried an electronic cigarette (yes; no; don’t know). This was used to determine e-cigarette trial status (tried prior to wave 1; tried between wave 1 and 3; never tried). Perceived relative harm was rated at each wave using the question: “Do you think electronic cigarettes are more harmful than regular cigarettes, less harmful, or are they equally harmful to health? (a) more harmful than regular cigarettes; (b) equally harmful; (c) less harmful than regular cigarettes; (d) don’t know”. For analysis, the response options were dichotomised into less harmful (c) and all other, inaccurate, responses (a, b and d).

Current e-cigarette use among those who had tried an e-cigarette was determined using the question: “How often, if at all, do you currently use an electronic cigarette? (a) daily; (b) less than daily, but at least once a week; (c) less than weekly, but at least once a month; (d) less than monthly; (e) not at all; (f) don’t know”. For analysis, responses were collapsed into any current use (a–d) and non-use (e); (f) was excluded. Smoking status was determined using the question: “Which of the following best applies to you? (a) I smoke cigarettes (including hand-rolled) everyday; (b) I smoke cigarettes (including hand-rolled) but not every day; (c) I do not smoke cigarettes at all but I do smoke tobacco of some kind (e.g. pipe or cigar); (d) I have stopped smoking completely in the last year; (e) I stopped smoking more than a year ago (at waves 2 and 3 only); (f) Don’t know/couldn’t say”. For analysis, responses were collapsed into current smoker (a–c) or ex-smoker (d and e); (f) was excluded. Based on responses across the three waves, change in smoking status across the waves was categorised as: smoker throughout; ex-smoker throughout; relapsed to smoking; stopped smoking. For *n* = 33 (2.7%) with more than one change in smoking status over the three waves, change from wave 2 to 3 was used to predict perceived relative harm at wave 3 (aim 2).

### 2.3 Analysis

Characteristics of those successfully followed up and those lost to follow-up were compared using chi-square statistics and a *t*-test for age.

To address aim 1, proportions of responses about perceived relative harm across the three waves were analysed descriptively. A Friedman test was used to assess change across all three waves, followed by McNemar tests for comparison between two waves. In a sensitivity analysis, the analyses were repeated with the exclusion of those responding ‘don’t know’ to the question about perceived relative harm.

To address aim 2, bivariate and multivariable logistic regressions were used to assess predictors of perceived relative harm at wave 3. Predictors included in the regressions models were perceived relative harm at waves 1 and 2, gender, age (grouped), education and income at wave 1, change in smoking across the waves and e-cigarette trial status.

And to address aim 3, bivariate and multivariable logistic regressions were used to assess if perceived relative harm among non-e-cigarette users at wave 1 predicted use of e-cigarettes at wave 2; multivariable regression adjusted for wave 1 gender, age (grouped), education, income and smoking status. Analyses were repeated to assess if perceived relative harm among non-e-cigarette users at wave 2 predicted use of e-cigarettes at wave 3; the sample available for these analysis was much smaller (*n* = 364).

## 3 Results

### 3.1 Attrition and sample characteristics

Compared with respondents who were lost to follow-up between wave 1 and wave 2, respondents who were followed up at wave 2 were older, less likely to be female, to have any higher education, to have an annual income >£30,000, to perceive e-cigarettes as less harmful than cigarettes and to have tried e-cigarettes at wave 1; smoking status did not differ (Table 1). Comparison of those lost to follow-up with those followed up at wave 3 showed very similar results to the attrition analysis for wave 2 with the exception of income which no longer differed (*p* = 0.63). Key sample demographics are presented for each analysis in Table 2, Table 3.

### 3.2 Aim 1: Perceived relative harm over time

**Fig. 1:**
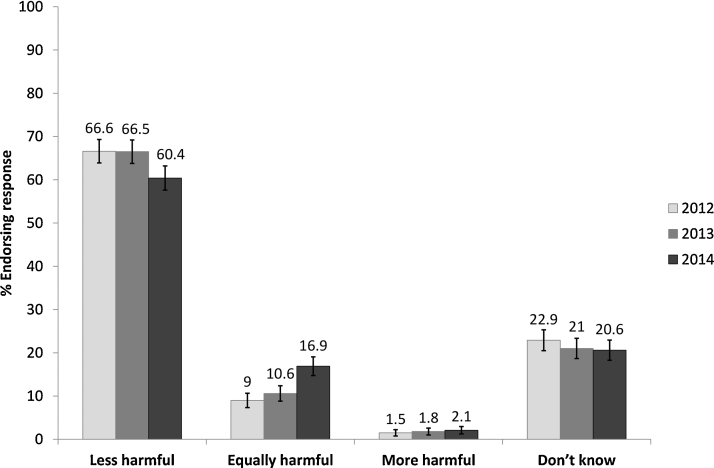
Perceived harm of electronic cigarettes compared with cigarettes, *n* = 1204. Error bars: 95% confidence intervals using the Wilson procedure (Newcombe, 1998).

At all three waves, e-cigarettes were perceived as less harmful than cigarettes by the majority of respondents (Fig. 1). However, perceived relative harm changed across the three waves (*χ*^2^ = 20.67, *p* < 0.001); there was no significant change from wave 1 to wave 2 (*p* > 0.99), but there was a significant decrease from wave 2 to 3 in the proportion thinking that e-cigarettes were less harmful than cigarettes (*χ*^2^ = 16.55, *p* < 0.001). In the sensitivity analysis excluding don’t know responses, the proportion perceiving e-cigarettes as less harmful than cigarettes decreased in a similar fashion (*n* = 705, wave 1: 86.0%; wave 2: 86.4%; wave 3: 78.2%; wave 2 versus wave 3: *χ*^2^ = 28.01, *p* < 0.001).

### 3.3 Aim 2: Predictors of perceived relative harm

In unadjusted and adjusted analysis, previous perception of e-cigarettes as less harmful, having tried e-cigarettes prior to or during the study period, and having stopped smoking during the study period were associated with greater odds for perceiving e-cigarettes as less harmful than cigarettes at wave 3 (Table 2). Being aged 55 years or over was associated with greater odds for perceiving e-cigarettes as less harmful than cigarettes at wave 3 only when adjusting for other predictors.

### 3.4 Aim 3: Prediction of subsequent use

Among those not using e-cigarettes at wave 1, 23% used e-cigarettes at wave 2. As described in the methods section, ‘use’ included any frequency of use but not those who reported trial but no current use. Perceiving e-cigarettes to be less harmful than cigarettes at wave 1 predicted use at wave 2 (OR = 1.43, 95% CI: 1.12 to 1.84, *p* = 0.005). This association remained similar in adjusted analysis which also indicated that at wave 2, women and wave 1 smokers were more likely to report use for the first time (Table 3).

In the smaller sample available for prediction of use at wave 3, the association of perceived relative harm with subsequent use was weakened. Among those not using e-cigarettes at wave 2, 37.9% of those who perceived e-cigarettes as less harmful than cigarettes reported any use at wave 3, compared with 34.7% of those with any other response to perceived relative harm (OR = 1.15, 95% CI: 0.73 to 1.81, *p* = 0.56). Results were similar when adjusting for wave 2 characteristics (OR = 1.11, 95% CI: 0.70 to 1.78, *p* = 0.66).

## 4 Discussion

In a longitudinal survey of smokers and ex-smokers in Great Britain with annual waves in 2012, 2013 and 2014, there was a decrease in the proportion of respondents accurately perceiving e-cigarettes to be less harmful than cigarettes between 2013 and 2014. This perception of e-cigarettes as less harmful was more likely among older respondents, those who had previously perceived e-cigarettes to be less harmful than cigarettes, had tried e-cigarettes, and those who had successfully stopped smoking during the study period. Accurately perceiving e-cigarettes as less harmful than cigarettes predicted subsequent use of e-cigarettes among respondents who had not previously tried an e-cigarette, even when adjusting for demographics and smoking status.

Limitations of this study include the loss to follow-up, contributing to small sample sizes for some subgroups such as ex-smokers and relapsed smokers, resulting in reduced confidence in the results for these groups. Also, some respondents may have changed their smoking status more often than reflected in the annual surveys. Another limitation is differential attrition, potentially reducing generalisabilty to younger, female and more highly educated smokers or recent ex-smokers. Attrition also differed with perceived relative harm at baseline such that those followed up were less likely to perceive e-cigarettes as less harmful at baseline than those lost to follow-up. Differential attrition therefore reduced the chances of detecting a decrease in the perception of e-cigarettes as less harmful than cigarettes. The change over time found in the present data may thus have been larger with higher follow-up rates.

Confidence in the present finding that perceived harm increased is strengthened by its agreement with findings from other surveys from the UK and the US (Action on Smoking and Health (ASH), 2015, Eastwood et al., 2015, Tan and Bigman, 2014). Cross-sectional annual surveys of representative samples of smoking and non-smoking adolescents and adults in Great Britain also found significant increases in the proportion who considered e-cigarettes to cause about the same level of harm to the user as cigarettes from 2013 to 2015 (Action on Smoking and Health (ASH), 2015, Eastwood et al., 2015). In an overview of US surveys, the proportion of US smokers aware of e-cigarettes who perceived them to be less harmful than cigarettes was smaller in a survey conducted in 2012/2013 than in surveys conducted in 2010 (Tan and Bigman, 2014).

Potential harms of e-cigarettes have been discussed both in relation to users and bystanders. The question used in the present study asked about the relative harmfulness of e-cigarettes in general and did not differentiate between harm to the user or others and did not specifically ask about the harmfulness of switching to, or dual use of e-cigarettes alongside cigarettes, all of which may have led to different findings. However, similar measures have been utilised in previous surveys on relative harm of nicotine containing products, increasing the comparability of these findings (Amrock et al., 2015, Borland et al., 2011, Eastwood et al., 2015, Richardson et al., 2014b, Tan and Bigman, 2014). As those studies, the present study was not designed to test a model of behaviour change; internal and external factors other than perceived harm will have influenced whether a respondent used an e-cigarette.

The major strength of this study is that it is the first longitudinal study with detailed information on e-cigarette use and perception of e-cigarettes that followed respondents over two years, which allowed analysis of factors that influenced subsequent uptake of e-cigarettes among those who had not used them.

The proportion of current smokers in the 2014 wave of this survey who perceived e-cigarettes as less harmful was about 58%. In comparison, a monthly household survey of the population in England, estimated that only 44.1% of current smokers surveyed between November, 2014 and March, 2015 believed that e-cigarettes were less harmful than cigarettes (Brown et al., 2015). The difference may be related to differences in recruitment (online versus face-to-face), design (longitudinal vs. repeated cross-sectional) or the sampling of different countries (all of Great Britain vs. England only). We have no information about why perception of relative harm changed, and can only speculate that this may be due to a predominance of reports and discussions focusing on the risks of e-cigarettes without comparison to the much greater risks posed by cigarettes.

The finding that perceptions predicted subsequent use extends previous cross-sectional findings showing associations of e-cigarette use and harm perception (Adkison et al., 2013, Amrock et al., 2015, Pokhrel et al., 2015, Richardson et al., 2014b). The present study further extends these findings by showing that a change in behaviour, i.e. initiating trial of e-cigarettes or stopping smoking affected subsequent harm perception. Findings are in line with previous associations of risk perception and use found for smokeless tobacco and nicotine replacement therapy (O’Connor et al., 2007, Shiffman et al., 2008). An interesting additional finding of the present study not related to the research questions is that smokers were more likely to take up e-cigarettes during the subsequent year than non-users who were already ex-smokers at baseline, suggesting a reduced risk for existing ex-smokers to initiate e-cigarette use.

The present findings suggest that misperceptions of relative harm may impede switching. If e-cigarette use was avoided in favour of stopping nicotine use entirely, smokers’ health would benefit, however, the risk is that smokers continue smoking instead. One recent US survey for example found that a substantial proportion of respondents who had tried an e-cigarette reported that health concerns had led them to stop e-cigarette use; all continued smoking instead (Biener and Hargraves, 2015). Importantly, all smokers, including e-cigarette users who continue smoking cigarettes should be given advice and be supported to stop smoking cigarettes using the best available evidence (e.g., Aveyard et al., 2012, Fiore et al., 2008, West et al., 2000).

## 5 Conclusion

Among a cohort of smokers and ex-smokers, accurately perceiving e-cigarettes as less harmful than smoking predicted subsequent e-cigarette use in never-users; this perception declined over time. Clear and balanced information on the relative harm of e-cigarettes and cigarettes is needed.

## Funding and role of the funding source

All authors are part of the UK Centre for Tobacco and Alcohol Studies, a UK Clinical Research Collaboration Public Health Research: Centre of Excellence. Funding from the Medical Research Council, British Heart Foundation, Cancer Research UK, Economic and Social Research Council and the National Institute for Health Research under the auspices of the UK Clinical Research Collaboration is gratefully acknowledged (MR/K023195/1). Cancer Research UK (C25586/A19540) have partly funded the work for this manuscript. JB's post is funded by a fellowship from the UK Society for the Study of Addiction. The funders played no role in the study design, collection, analysis and interpretation of the data, in the writing of the manuscript and in the decision to submit this manuscript for publication.

## Contributors

AM conceived of the survey in collaboration with Professor Robert West and with input on the design from JB, SH and LB. All authors contributed to the statistical analysis plan. LB conducted the analysis, and drafted and revised the manuscript. SH, JB & AM provided significant input in re-drafting. All authors contributed to and have approved the final manuscript.

## Conflict of interest statement

JB has received an unrestricted grant from Pfizer. LB, SH and AM have no relationships with companies that might have an interest in the submitted work. There are no other financial relationships with any organizations that might have an interest in the submitted work, particularly electronic cigarette or tobacco companies, and there are no other relationships or activities that could appear to have influenced the submitted work.

## Figures and Tables

**Table 1:** Wave 1 characteristics of those followed up at wave 2 and those lost to follow-up.

	Followed up	Lost to follow-up	Comparison
Age, mean, standard deviation	46.4, 15.5		*t* = 13.3, *p* < 0.001
Female (%)	41.8	51.7	*χ*^2^ = 48.9, *p* < 0.001
Some higher education (%)	35.4	36.9	*χ*^2^ = 11.6, *p* = 0.003
Annual income >£30,000 (%)	37.9	41.1	*χ*^2^ = 4.9, *p* = 0.027
Tried e-cigarette (%)	35.2	41.0	*χ*^2^ = 15.9, *p* < 0.001
Perceived e-cigarettes as less harmful (%)	66.8	70.7	*χ*^2^ = 7.8, *p* = 0.006
Current smoker (%)	87.1	86.6	*χ*^2^ = 0.3, *p* = 0.60

**Table 2:** Wave 1 (2012) and wave 2 (2013) predictors of perceiving e-cigarettes as less harmful than regular cigarettes at wave 3 (2014), *n* = 1204.

	*n* (% of 1204)	% e-cigarettes less harmful than cigarettes, 2014	Unadjusted (bivariate) analysis				Adjusted (multivariable) analysis			
			Odds ratio	95% CI			Odds ratio	95% CI		
				Lower	Upper	*p*-Value		Lower	Upper	*p*-Value
E-cigarettes perceived as less harmful than cigarettes, 2012	802 (66.6)	70.1	**3.36**	**2.62**	**4.32**	**<0.001**	**1.93**	**1.45**	**2.57**	**<0.001**
E-cigarettes perceived as less harmful than cigarettes, 2013	801 (66.5)	75.3	**6.85**	**5.25**	**8.94**	**<0.001**	**5.33**	**4.00**	**7.11**	**<0.001**
Male (referent)	714 (59.3)	61.8	1	1	1	ref	1	1	1	ref
Female	490 (40.7)	58.4	0.87	0.69	1.1	0.24	0.77	0.59	1.01	0.062
Age, 2012										**0.004**
18-24 (referent)	103 (8.6)	60.2	1	1	1	ref	1	1	1	ref
25-39	275 (22.8)	55.6	0.83	0.52	1.32	0.43	0.96	0.57	1.61	0.87
40-54	390 (32.4)	60.3	1.00	0.64	1.56	0.99	1.36	0.82	2.24	0.23
55 and over	436 (36.2)	63.5	1.15	0.74	1.79	0.53	**1.82**	**1.10**	**3.01**	**0.020**
Education, 2012										0.22
No higher education (referent)	765 (63.5)	60.8	1	1	1	ref	1	1	1	ref
Some higher education	418 (34.7)	60.5	0.99	0.78	1.26	0.93	0.90	0.68	1.21	0.487
Don’t know/prefer not to say	21 (1.7)	42.9	0.48	0.20	1.16	0.10	0.44	0.17	1.18	0.10
Annual income, 2012										0.71
Up to £30,000 (referent)	675 (56.1)	60.0	1	1	1	ref	1	1	1	ref
Over £30,000	417 (34.6)	63.3	1.15	0.89	1.48	0.28	1.09	0.81	1.46	0.59
Don’t know/prefer not to say	112 (9.3)	51.8	0.72	0.48	1.07	0.10	0.88	0.55	1.42	0.61
E-cigarette status during study period										**<0.001**
Never tried (referent)	340 (28.2)	45.0	1	1	1	ref	1	1	1	ref
Reported trial in 2012	451 (37.5)	69.8	**2.83**	**2.11**	**3.80**	**<0.001**	**2.09**	**1.49**	**2.93**	**<0.001**
Tried after 2012	413 (34.3)	62.7	**2.06**	**1.54**	**2.75**	**<0.001**	**1.78**	**1.27**	**2.51**	**0.001**
Smoking status during study period										**0.049**
Smoker throughout (referent)	893 (74.2)	58.6	1	1	1	ref	1	1	1	ref
Ex-smoker throughout	43 (3.6)	69.8	1.63	0.84	3.17	0.15	1.95	0.88	4.32	0.100
Relapsed to smoking	67 (5.6)	53.7	0.82	0.50	1.35	0.44	0.87	0.49	1.54	0.62
Stopped smoking	201 (16.7)	68.7	**1.55**	**1.12**	**2.15**	**0.009**	**1.53**	**1.06**	**2.20**	**0.025**

*Note:* Associations with *p* < 0.05 in bold

**Table 3:** Wave 1 (2012) predictors of wave 2 (2013) e-cigarette use in those not using e-cigarettes at wave 1, *n* = 1588.

	N (% of 1588)	% using e-cigarettes, 2013	Adjusted Odds ratio	95% Confidence interval		
				Lower	Upper	*p*-Value
E-cigarettes perceived at least as harmful as cigarettes or don’t know (referent)	590 (37.2)	19.2	1	**1**	**1**	ref
E-cigarettes perceived as less harmful than cigarettes	998 (62.8)	25.4	**1.39**	**1.08**	**1.80**	**0.011**
Male (referent)	930 (58.6)	19.7	1	1	1	ref
Female	658 (41.4)	27.8	**1.55**	**1.21**	**1.97**	**<0.001**
Age						**0.028**
18–24 (referent)	149 (9.4)	26.2	1	1	1	ref
25–39	398 (25.1)	27.6	1.17	0.76	1.81	0.48
40–54	498 (31.4)	22.7	0.89	0.58	1.36	0.58
55 and over	543 (34.2)	19.2	0.72	0.47	1.11	0.14
Education						
No higher education (referent)	1020 (64.2)	22.9	1	1	1	ref
Some higher education	531 (33.4)	22.8	0.94	0.72	1.22	0.64
Don’t know/prefer not to say	37 (2.3)	29.7	1.71	0.81	3.60	0.16
Annual income						
Up to £30,000 (referent)	897 (56.5)	24.2	1	1	1	ref
Over £30,000	518 (32.6)	22.2	0.91	0.69	1.20	0.51
Don’t know/prefer not to say	173 (10.9)	19.7	0.82	0.54	1.24	0.35
Smoking status						
Ex-smoker (referent)	209 (13.2)	11.5	1	1	1	ref
Current smoker	1379 (86.8)	24.8	**2.61**	**1.67**	**4.07**	**<0.001**

*Notes:* Associations with *p* < 0.05 in bold. ‘% using e-cigarettes’ includes any frequency of use but not those who reported trial but no current use.
